# Dataset providing boundary conditions of an experimental test bench to numerically investigate flame wall interactions using CFD

**DOI:** 10.1016/j.dib.2022.108617

**Published:** 2022-09-17

**Authors:** Lukas Fischer, Rahand Dalshad, Paola Breda, Michael Pfitzner

**Affiliations:** University of the Bundeswehr Munich, Department of Aerospace Engineering, Institute of Thermodynamics, Werner-Heisenberg-Weg 39, Neubiberg, 85579, Germany

**Keywords:** Autoignition delay, Wall heat flux, Secondary reactions, Methane combustion, CFD, Boundary condition

## Abstract

The data provided allows other research groups to numerically investigate the flame wall interactions of an experimental test bench at the University of the Bundeswehr Munich. Numerical simulations can then be compared to the experimental results to test new models. Three data sets are available. The first data set contains the inflow boundary conditions created by the burner into the test section. The fields of interest are the velocity and RMS-velocity fields. Large-Eddy-Simulation (LES) using ANSYS Fluent was used to collect this data. The second data set provides the inflow boundary condition from the fuel injector into the test section using methane at a momentum ratio of I = 10. LES using OpenFOAM was used to create this data. The third data set provides the temperature distribution at the wall of the test section when injecting methane at a momentum ratio of I = 10. The temperature is provided along the wall ranging from +/- 25 mm in the lateral and 0 - 220 mm in the axial direction. The data was derived from wall-embedded thermocouples by applying the inverse heat conduction method using MATLAB and COMSOL.

**Specifications Table**

**Table:** 

Subject	Aerospace Engineering
Specific subject area:	Flame wall interactions in gas turbines
Type of data:	Table
How the data were acquired:	Dataset1: LES with OpenFOAM; Dataset2: LES with ANSYS Fluent, Dataset3: Inverse Heat Conduction Method from thermocouple signals. MATLAB and COMSOL for post-processing
Data format:	Raw
Description of data collection:	SI-units are used in the dataset. The data is not normalized. dataset_1_testsection_inlet: rmse = rms = root mean square dataset_2_hole_fuel_injection: U:0 = U = x_velocity U:1 = V = y_velocity U:2 = W = z_velocity Points:0 = x_coordinate Points:1 = y_coordinate Points:2 = z_coordinate dataset_3_testsection_temperature: x, y, z: coordinates T: Static temperature
Data source location:	• *Institution:* University of the Bundeswehr Munich • *City/Town/Region: Neubiberg, Bavaria* • *Country: Germany*
Data accessibility:	Repository name: Mendeley Data Data identification number: DOI: https://doi.org/10.17632/jtmk3pg36k.1
Related research article:	*L. Fischer, P. Breda, R. Dalshad, M. Pfitzner, Numerical characterization of a novel test bench featuring CH4 secondary reactions. Aerospace Science and Technology (2021) - Volume 119*, https://doi.org/10.1016/j.ast.2021.107203

## Value of the Data

• The boundary conditions can be used to validate numerical models by comparing the results to published experimental results by Dalshad et al. [2], [3].
• Other researching groups can benefit from the data because the data is not available otherwise.
• The boundary conditions can be applied to new CFD simulations by directly interpolating those onto the patches of the CFD domain.
• High numerical effort to reproduce the boundary conditions in the fluid domain is omitted.

## 1 Data Description

Dataset1: This dataset provides the boundary condition created by the burner upstream of the fuel injectors. Raw data is available as a table with the x, y, z coordinates of the test section wall with units in meter. The time averaged temperature is given in Kelvin. The time averaged velocity and RMS-velocity is given as well. Units are given in meter per second. In addition, the time averaged (static) pressure field is given in Pascal.

Dataset2: This dataset provides the boundary condition of the fuel injector outlet just upstream of the test section. Raw data is available as a table with the x, y, z coordinates of the test section wall with units in meter. The temperature is given in Kelvin. The velocity is given as well. Units are given in meter per second. The (static) pressure field is given in Pascal. In addition, the mass fractions of the species ($CH_{4}$, $CO_{2}$, $H_{2}O$, $N_{2}$, $O_{2}$) are given which are without unit.

Dataset3: This dataset provides the boundary condition of the temperature at the test section wall. Raw data is available as a table with the x, y, z coordinates of the test section wall with units in meter. Moreover, the temperature at the wall is given in Kelvin.

## 2 Experimental Design, Materials and Methods

Dataset1: The fluid domain of the experimental setup of the burner was meshed using ANSYS ICEM CFD. An unstructured mesh was created with 10 inflation layers to resolve the boundary layer and to allow for $y^{+}<1$. The mesh consisted of 6 million elements. The mesh was smoothed. ANSYS Fluent was chosen as the solver for the flow field. The realizable k-ε model was chosen for the preliminary RANS simulation on a coarse mesh. The WALE turbulence model was chosen for the LES simulations. A massflow of 768 g/min was assigned to the inlet of the burner. The (static) temperature at the inlet was set to 1430 K. The 2-D contour of the (static) temperature along the test sections wall was applied (mapped from Dataset3). All other walls were assumed to be adiabatic. The no-slip condition was applied to all walls. The pressure and velocity fields were solved using the coupled scheme. The gradients were discretized using the Green-Gauss Node based scheme. For the pressure the second order scheme was chosen. The bounded central difference scheme was chosen for the momentum equations. The bounded second order implicit scheme was chosen for the temporal integration. ANSYS Fluent is capable of using the adaptive time stepping method to keep a maximum CFL number of 0.9 for each integration step. The LES was started from the RANS solution. Before time averaging over 3.5 through flows, 4 through flow times were allowed to remove the initial transient solution. The data of dataset1 was then extracted from a plane just upstream of the location where the fuel injectors would be located. For more details the reader is referred to the related research article [4].

Dataset2: The fluid domain of the fuel injector was created by using ANSYS ICEM CFD. A block structured approach was chosen containing 1.1 million cells with a wall resolution of $y^{+}<1$ for most of the wall. Near the inlet and outlet of the fuel injector some areas have $y^{+}$ values between 4-6. OpenFOAM 6 was chosen to solve the equation system using the *reactingFoam* solver. The WALE turbulence model was chosen for the LES. Pure second order schemes were applied, namely the central difference scheme (Gauss Linear) for the divergence terms and the second order backward Euler scheme (backward) for the temporal integration. The iterative PIMPLE algorithm was applied with 2 loops to lower the initial residuals by several orders of magnitude for each time step. The adaptive time stepping method was applied to allow for a maximum CFL number of 0.6. The resulting time step was of the order $1\cdot 10^{-8}\;s$. No time average was taken because after 15 through flows the flow field at the exit of the fuel injector showed a laminar profile. Probe points at this location showed a variation of the velocity below 0.02% during this period. The data of dataset2 was then extracted from a plane at the outlet of the fuel injector just upstream of the test section. For more details the reader is referred to the related research article [4].

Dataset3: The temperature of the test section wall was determined when methane was injected into the test section at a momentum ratio of I=10. The static temperature in the mainflow was around 1600 K. The surface temperature is theoretically and numerically reconstructed from local temperature measurements within the test section wall. Generally, heat conduction problems are either of direct or inverse character. In direct cases, the thermal boundaries, which is a cause, are known and the temperature and heat flux distribution within the domain, which are the effects, are calculated using heat conduction theories. In inverse problems on the other hand, some effects are known and the cause is subject of interest. The inverse calculation is mathematically demanding and often unstable. Thus, the inverse method is reduced to an iterative process of direct calculation. In the current work, a number of thermocouples (TC) are installed within the test section wall and record the local temperatures transiently. Subsequently, the idea is to perform direct heat conduction simulations for the test section domain at each time step that the least square norm or so-called objective function S converges to a user-defined accuracy ε:

(1) $$S\left(Q\right)={\left[Y-T(Q)\right]}^{T}\left[Y-T(Q)\right]=\varepsilon$$

where Y is the vector of measured temperatures at each time step and T(Q) is the vector of iteratively determined temperature depending on the heat flux vector Q. The superscript T denotes the transpose form. The heat flux is applied to the surface of interest at the locations where the TC are projected onto. For the iterative determination of the heat flux vectors, the Levenberg-Marquardt method is used:

(2) $$Q^{k+1}=Q^{k}+{\left[{\left(J^{k}\right)}^{T}J^{k}+\mu^{k}\Omega^{k}\right]}^{-1}{\left(J^{k}\right)}^{T}\left[Y-T(Q^{k})\right]$$

where k indicates the iteration step, μ is a damping parameter for stabilization and Ω is a diagonal matrix calculated from $diag\left[{\left(J^{k}\right)}^{T}J^{k}\right]$. J is the Jacobian or sensitivity matrix, which basically describes how the temperature at each location of interest changes when the corresponding heat flux is altered:

(3) $$\mathrm{J(Q)}={\left[\frac{\partial T^{T}\left(Q\right)}{\partial Q}\right]}^{T}$$

The coefficients of the matrix are determined from a forward difference scheme by applying a heat flux at each thermocouple position successively and obtaining the temperature. For detailed explanation and implementation procedures, the reader is referred to [6]. The entire data acquisition and optimization post-processing is as follows: A number of thermocouples installed within the test section wall record the temperature at a sampling rate of 2.85 Hz during the experiment. To reduce noise and the sample rate, the data is filtered to 0.1 Hz. Then, a direct heat conduction simulation is setup for the test section wall in [1] and the known boundary conditions are assigned, such as the convective water-cooling. COMSOL is a commercial finite element tool with graphical user interface. The surface exposed to the hot gas is initially assigned with a zero heat flux. Within the Matlab [5] domain, heat flux values according to equations Eq. (2) are determined and assigned to the hot gas surface. Interpolation schemes are used to generate profiles from the discrete heat flux values. Another direct simulation process is conducted and the residual according to Eq. (1) is checked. This procedure is iteratively performed until the objective function converges to a specified value, generally in the range of the standard deviation of the experimental temperature data (around 0.1 K). Afterwards, the optimization of the next time step is launched. Finally, a 3D solution of the wall test section is obtained. The 2D contour of the test section’s wall is extracted and provided in dataset3.

## Ethics Statements

The data does not involve human subjects, animal experiments and was not collected from social media platforms.

## CRediT authorship contribution statement

**Lukas Fischer:** Conceptualization, Software, Validation, Writing – original draft. **Rahand Dalshad:** Conceptualization, Software, Validation, Writing – original draft. **Paola Breda:** Conceptualization, Software, Validation, Writing – original draft. **Michael Pfitzner:** Supervision, Writing – review & editing.

## Declaration of Competing Interest

The authors declare the following financial interests/personal relationships which may be considered as potential competing interests.

## Data Availability

Dataset providing boundary conditions of an experimental test bench to numerically investigate flame wall interactions using CFD (Mendeley Data).

## References

[1] COMSOL (2019). Comsol Multiphysics®v. 5.5.

[2] Dalshad R., Sander T., Pfitzner M. (2021). Volume 3A: Combustion, Fuels, and Emissions.

[3] Dalshad R., Sander T., Pfitzner M. (2021).

[4] Fischer L., Breda P., Dalshad R., Pfitzner M. (2021). Numerical characterization of a novel test bench featuring secondary reactions of methane. Aerosp. Sci. Technol..

[5] MATLAB (2019). Version 9.7.0 (r2019b).

[6] Ozisik M.N. (2000).

